# Oxygen Therapy and Ventilatory Support

**DOI:** 10.1155/2017/2462818

**Published:** 2017-05-14

**Authors:** Wan-Jie Gu, Zhongheng Zhang, Sven Van Poucke

**Affiliations:** ^1^Department of Anesthesiology, Nanjing Drum Tower Hospital, Medical College of Nanjing University, Nanjing, China; ^2^Department of Emergency Medicine, Sir Run Run Shaw Hospital, Zhejiang University School of Medicine, Hangzhou 310016, China; ^3^Ziekenhuis Oost-Limburg, Genk, Belgium

When Joseph Priestley discovered oxygen in 1774, he found that air was a mixture of gases. He called one of these gases “dephlogisticated air,” to which later the chemist Antoine Lavoisier gave the name “oxygen” which made candles burn brighter and longer than ordinary air [1]. Until now, this discovery covers 2 essential features of oxygen which in clinical practice results in the use of oxygen as a drug. In clinical practice, oxygen can be life-saving by giving to patients with tissue hypoxemia. However, too much oxygenation can cause toxicity, which has been well described in the literature. Oxygen toxicity in central nervous system is termed Paul Bert effect and in the lung it is termed Lorrain Smith effect. The Paul Bert effect results in seizures from which the onset depends upon the partial pressure of oxygen in the breathing gas and exposure duration. This effect is well known among divers and in hyperbaric facilities. Respiratory side effects from oxygen are more frequently observed in critical care settings resulting in a restrictive policy not to administer more oxygen than required. In this context, however, it is important to have the formulae of oxygen content in mind while not just relying on the oxygen saturation values (arterial oxygen content = (Hb × 1.34 × SaO_2_) + (0.0031 × PaO_2_)). Additionally, NICU patients are potentially at risk for Terry's syndrome or retinopathy of prematurity, a disease of the eye when oxygen is administered for premature development of the lungs. Although oxygen is a natural molecule, the complexity of its interactions with human metabolism continues to be a subject for in-depth research [2].

In this thematic issue of oxygen therapy and ventilatory support (OTVS), several papers investigated and discussed some key aspects in the field of respiratory failure and ventilator support. J.-L. Vincent et al. published an article addressing the harmful effect of hyperoxia on critical illness. It has been reported that the effect of partial pressure of arterial oxygenation on mortality follows a quadratic function [3], and there is nadir at which the mortality rate is the lowest. Respiratory support can be performed by a variety of techniques, ranging from the simple oxygen therapy to complex extracorporeal membrane oxygenation (ECMO). In patients with ECMO, the mechanical ventilation usually plays an important role. The appropriate setting of the ventilator is of vital importance to provide oxygen supply to vital organs while avoiding lung injury. It is an art and science where, in most circumstances, there is a lack of high-level evidence from randomized controlled trials. Z. Zhang and colleagues discussed some important aspects on the use of mechanical ventilation in patients with ECMO. S. Ye and his colleagues investigated the effect of antipyretic therapy on mortality outcome in patients receiving mechanical ventilation. The study employed the MIMIC-II database as the source of data [4]. The database is freely available to the public and is the gold standard of the critical care big data. It provides in-hospital clinical data with high granularity, and data mining using such database can provide insights into complex interactions between disease severity, medical treatment, and procedures. Such complex interactions may not be feasible in conventional randomized controlled trials (RCTs). Also, traditional RCTs are designed to explore the biological efficacy of a certain intervention, which may not be applicable to real world settings where the clinical effectiveness is most highlighted. There is evidence that the results obtained from RCTs can be different from that obtained by observational studies [5].

*Wan-Jie Gu* *Wan-Jie Gu* *Zhongheng Zhang* *Zhongheng Zhang* *Sven Van Poucke* *Sven Van Poucke*
